# Adriamycin-resistant cells are significantly less fit than adriamycin-sensitive cells in cervical cancer

**DOI:** 10.1515/biol-2021-0004

**Published:** 2021-01-20

**Authors:** Min Qi, Lijuan Xie, Guihua Duan

**Affiliations:** Department of Radiology, The Third People’s Hospital of Kunming City, The Sixth Affiliated Hospital of Dali University, Kunming 650041, China; Department of Infection, First Affiliated Hospital of Kunming Medical University, Kunming 650332, China; Department of Gastroenterology, The First People’s Hospital of Yunnan Province, The Affiliated Hospital of Kunming University of Science and Technology, Kunming 650032, China

**Keywords:** adriamycin resistance, cervical cancer, fitness differences, tumor evolution

## Abstract

Adriamycin (ADR) is an important chemotherapy agent in many advanced cancers, but the emergence of drug resistance during treatment is a major limitation to its successful use. Recent studies have suggested that drug-resistant cells become less fit and their growth could be inhibited by parental cells without cytotoxic treatment. In this study, we examined the fitness differences between HeLa and HeLa/ADR cells. Compared with the parental cell line, HeLa/ADR cells showed significantly lower growth rates, both *in vitro* and *in vivo*. There was no difference in the apoptosis rate between them, but G1 arrest and reduced DNA synthesis were found in HeLa/ADR cells. Further study indicated that HeLa/ADR cells failed to compete for space and nutrition against parental cells *in vivo*. Taken together, we demonstrate that HeLa/ADR cells are less fit and their growth can be inhibited by parental cells in the absence of ADR; therefore, the maintenance of a certain amount of ADR-sensitive cells during treatment may facilitate the control of the development of ADR resistance.

## 1 Introduction

Adriamycin (ADR) is a valuable clinical antitumor agent and is routinely used in the treatment of several cancers [1,2]. However, in addition to problems with toxicity, the dominant growth of ADR-resistant cells after treatment is a key factor limiting its use. Multiple studies have indicated the appearance of resistant cells prior to the initiation of therapy [3,4,5,6]. There are findings that even provide evidence that resistant cells can both preexist and evolve from drug-sensitive cells [7,8], and that cancer therapies may eventually select for resistant cells and further promote their clonal expansion [3,9,10]. However, some models suggest that the development of drug-resistant cells may be delayed in the presence of existing dominant clones owing to the limited availability of nutrition and space [11,12,13].

Adaptive therapy was first introduced by Gatenby [11]. The principle of adaptive therapy is to achieve a fixed tumor size by maintaining a certain amount of drug-sensitive cells, which can suppress the growth of less fit, but drug-resistant cells. In this way, adaptive therapy could significantly extend patient progression-free survival in both the mathematical model and various preclinical models of breast cancer [12,14]. Their model found that when resistant cells emerged in an untreated tumor, they are commonly present in small quantities and their growth is inhibited by existing sensitive cells that are more fit because resistance mechanisms need to consume additional resources for growth. Accordingly, drug-resistant cells are less fit, and this has been partially confirmed in a breast cancer cell line [12,14]. Therefore, treatments designed to kill all drug-sensitive cells may abrogate this counterbalancing effect and actually promote more rapid outgrowth of resistant cells.

Currently, few studies have examined the fitness distinctions between ADR-resistant and ADR-sensitive cells. Gatenby et al. reported that ADR-sensitive breast cancer cells are more fit than resistant cells in glucose-restricted conditions, and patient survival could be increased under certain conditions by utilizing the competition between drug-resistant and drug-sensitive cells according to certain computational models [14]. However, more direct experimental evidence about the fitness deficit of ADR-resistant cells may have important future implications and is currently limited.

In this study, we revealed that the proliferation of HeLa cells was substantially faster than that of HeLa/ADR cells both *in vitro* and *in vivo*, and HeLa/ADR cells failed to occupy space when introduced at a one-to-one ratio with sensitive cells *in vivo*. Our data provides a direct evidence that ADR-sensitive HeLa cells are significantly more fit than ADR-resistant HeLa cells, and adaptive strategy may have important implications in the treatment of cervical tumor.

## 2 Materials and methods

### 2.1 Cell culture

HeLa cell line was purchased from the Type Culture Collection of the Chinese Academy of Sciences, Shanghai, China. RFP-tagged HeLa cell line was derived via the lentiviral transduction. HeLa/ADR cell line was induced by sustained exposure of HeLa cells to incremental concentrations of ADR (KeyGEN BioTECH). The higher drug concentration was employed after the cells got into a steady growth period. IC_50_ was calculated by GraphPad Prism. In order to maintain the resistant phenotype, HeLa/ADR cell line was maintained in the presence of 90 ng/mL ADR until 1 week before experiments. All cell lines were cultured in RPMI 1640 (Invitrogen) supplemented with 10% FBS (Biological Industries, BI) under a 5% CO_2_ environment. All cell lines were authenticated by short tandem repeat profiling analysis.

### 2.2 Cell proliferation analysis

Cells were seeded onto 12-well plates at 1 × 10^4^ cells per well and incubated with 1 mL of media. Cells were counted daily by Cell Counter (Scepter 2.0, Millipore) for 9 days.

### 2.3 Cell viability assay

HeLa and HeLa/ADR cell lines were seeded onto 96-well plates at 3 × 10^3^ cells per well. After 24 h, growth media were exchanged for media containing different concentrations of ADR (0, 12.5, 25, 50, 100, 200, and 400 ng/mL). Cell viability was analyzed after 48 h by CCK8 (Dojindo) according to the manufacturer’s instruction.

### 2.4 Colony-formation assay

Cells were seeded onto 6-well plates at 500–1,000 cells per well and incubated with 2 mL of media with or without 50 ng/mL ADR for 14 days. Cells were fixed with 70% methanol for 10 min and stained with 0.5% crystal violet for 20 min. Colonies of more than 50 cells were counted under a microscope.

### 2.5 EdU assay

Cells were labeled using the Click-iT® Plus EdU (5-ethynyl-2′-deoxyuridine) Imaging Kit (Invitrogen) according to the manufacturer’s protocols. The ratio of EdU-positive cells was evaluated from three randomly selected sample regions by counting 500–1,000 cells per field using the ImageJ software (1.48 v).

### 2.6 Analysis of cell cycle distribution and apoptosis

Cell cycle analysis was performed using the Cycletest™ Plus DNA Reagent Kit (BD Bioscience) based on manufacturer’s instruction; then cells were analyzed by flow cytometry. Apoptosis rate *in vitro* was performed using the Annexin V-FITC apoptosis detection kit (BD Bioscience) based on manufacturer’s instruction; then cells were analyzed by flow cytometry.

### 2.7 Xenograft experiments

Male Nu/Nu mice of 4 weeks old were purchased from Vital River Laboratories. 10^6^ cells (total cell number was 2 × 10^6^ for the group which contained both RFP-tagged HeLa cells and HeLa/ADR cells) were suspended in 0.2 mL of RPMI 1640 supplemented with 50% Matrigel (BD Biosciences) before subcutaneous implantation into the flank region of each mice. *n* = 5 for HeLa group, *n* = 6 for mixed group, and *n* = 12 for HeLa/ADR group; HeLa/ADR cells were implanted into each flank of the six mice. Tumor volumes were monitored using electronic calipers twice a week; when the tumor volume reached 1,000–2,000 mm^3^, the mice were sacrificed. Tumor volumes were calculated using the following formula: 1/2 × length × width^2^. Length indicated the longest diameter of tumor.


**Ethical approval:** The research related to animal use has been complied with all the relevant national regulations and institutional policies for the care and use of animals and has been approved by the Medical Ethics Review Committee of the First People’s Hospital of Yunnan Province (Kunming, China).

### 2.8 Immunohistochemistry

Tumor tissues were fixed in 10% formalin (Sigma) at room temperature and embedded in paraffin. Paraffin-embedded samples were then processed for immunohistochemistry; Ki67 (1:100, 0.2 mg/mL, ab8191; Abcam) was used as a measure of cell proliferation. Scoring for the expression of Ki67 was performed as follows: the percentage of Ki67^+^ cells was calculated from three randomly selected regions of the samples by counting an average of 1,600–2,000 cells per slide using the ImageJ software.

### 2.9 RFP ratio analyses and terminal deoxynucleotidyl transferase dUTP nick-end labeling (TUNEL) assay

Tumor samples were frozen in liquid nitrogen for RFP ratio assay. 5 µm sections of frozen samples were prepared by freezing microtome, and cell nucleus was stained with DAPI. TUNEL assay was determined by the *in situ* cell death detection kit (Roche) according to the manufacturer’s protocols. The percentages of RFP-positive and TUNEL-positive cells were calculated from three randomly selected regions of the xenografts by counting an average of 1,600–2,000 cells per slide using the ImageJ software.

### 2.10 Statistical analyses

All the statistical analyses were performed using GraphPad Prism 6.0. All the experiments were repeated at least three times. Unless otherwise indicated, all experiments data were expressed as mean ± SD of triplicate wells of a representative experiment. Difference in tumor formation rate was evaluated by the Chi-square test. Differences between treatments were evaluated by Student’s *t* test. Differences were considered statistically significant when *P* < 0.05 (*P < 0.05, **P < 0.01, and ***P < 0.001).

## 3 Results

### 3.1 The development of HeLa cells is significantly faster than that of HeLa/ADR cells *in vitro*

**Figure 1:**
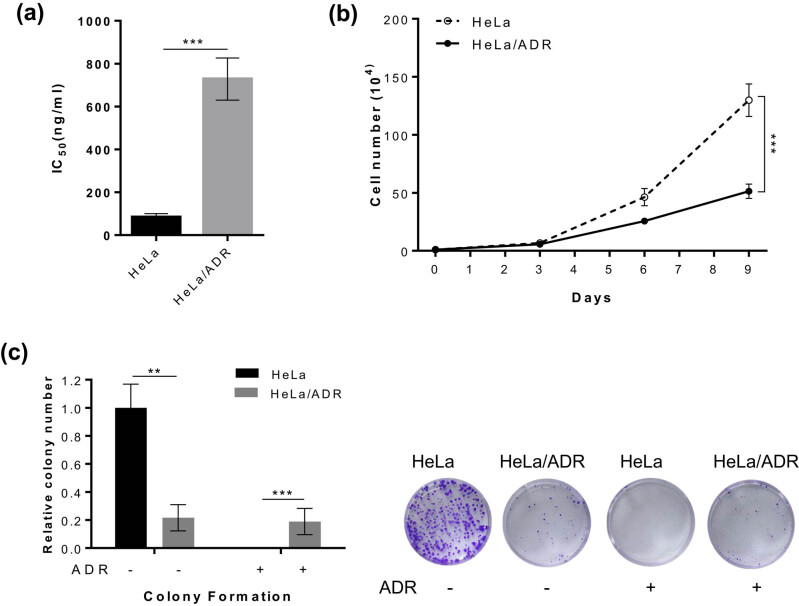
The growth of ADR-sensitive cells is substantially faster than that of ADR-resistant cells *in vitro.* (a) The IC_50_ values for HeLa and HeLa/ADR. (b) Growth curve of both cell lines in the absence of ADR. (c) The colony-formation assay was performed in HeLa and HeLa/ADR under conditions indicated. ADR (50 ng/mL) was added to the medium after 24 h. The clonogenic growth of HeLa/ADR cell line was not impacted by ADR, whereas the clonogenic growth of HeLa cell line was completely suppressed (*P < 0.05, **P < 0.01, and ***P < 0.001).

The IC_50_ values for ADR of both cell lines were evaluated, and the IC_50_ value of HeLa/ADR cells was almost ten times higher than that of HeLa cells (Figure 1a). Next, we evaluated the growth of both cell lines *in vitro* (Figure 1b). The growth rate of the HeLa cell line was faster than that of the HeLa/ADR cell line. In the colony-formation assay, more colonies formed in the HeLa cell line than the HeLa/ADR cell line, but the clonogenic growth of HeLa cell line was completely suppressed by ADR (Figure 1c); however, the clonogenic growth of HeLa/ADR cell line was not impacted. These results showed that the growth of the HeLa/ADR cell line was apparently slower than that of its parental cell line without drug treatment.

### 3.2 The slower growth rate of HeLa/ADR cells is due to reduced proliferation

**Figure 2:**
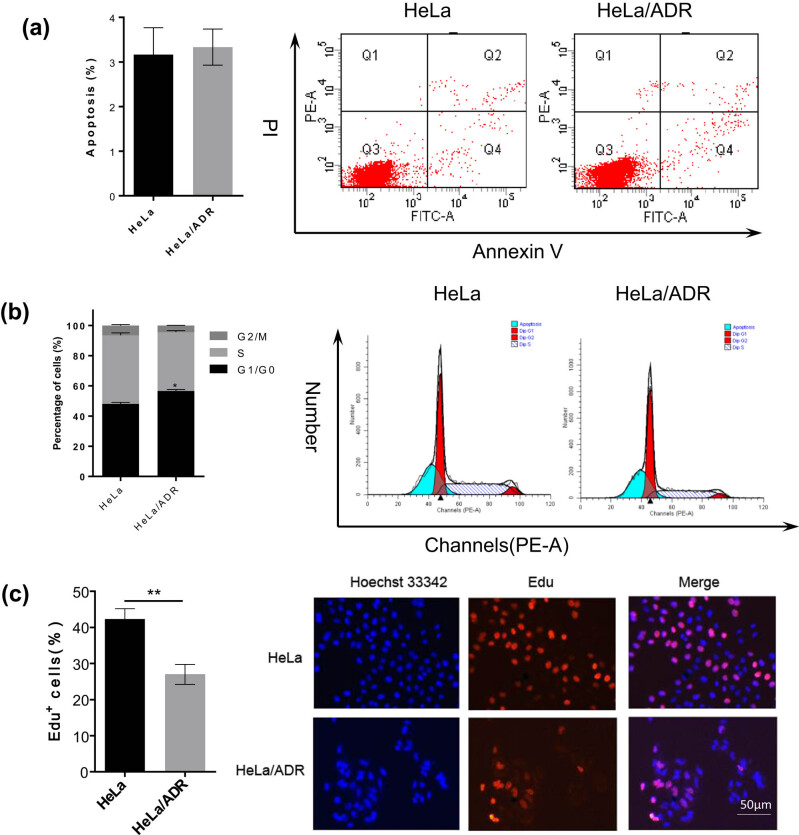
The slower growth rate of HeLa/ADR cells is owing to reduced proliferation. (a) Apoptosis of HeLa and HeLa/ADR cells under different conditions. The medium was exchanged after 24 h. Apoptosis analysis was performed 72 h after medium substitution. (b) Cell cycle analysis of HeLa and HeLa/ADR cells. The medium was exchanged after 24 h. The cell cycle analysis of both cell lines was performed 48 h after medium substitution. (c) EdU assay of HeLa and HeLa/ADR cells. The medium was replaced after 24 h. The EdU-positive cells were analyzed 48 h after medium substitution (*P < 0.05, **P < 0.01, and ***P < 0.001).

Next, we further investigated the reasons for the slower growth rate of the HeLa/ADR cells compared with HeLa cells. First, we revealed that the apoptosis rate was similar in both cell lines (Figure 2a), but a significant increase in G1 arrest was observed in HeLa/ADR cells compared with HeLa cells (Figure 2b). Consistent with the cell cycle distribution results, an EdU proliferation assay showed that HeLa/ADR cells had significantly reduced DNA synthesis compared with that of HeLa cells (Figure 2c). These results demonstrated that the lower growth rate of HeLa/ADR cells was caused by a reduced proliferation rate and not by an increased apoptosis rate.

### 3.3 HeLa cells are more fit than HeLa/ADR cells *in vivo*

**Figure 3:**
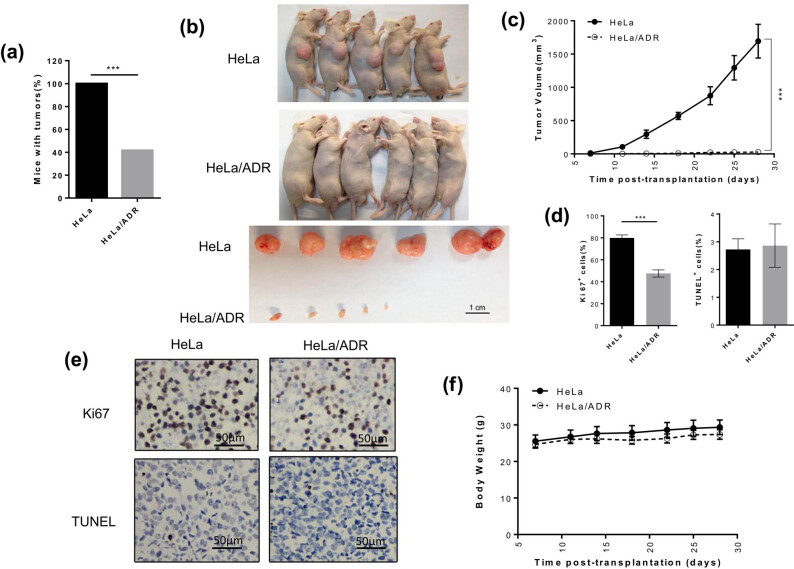
ADR-resistant cells exhibit poor adaptability compared with parental cells *in vivo.* (a) Both cell lines were grafted and monitored for tumor formation over 1 month; *n* = 5 for HeLa group and *n* = 12 for HeLa/ADR group. HeLa/ADR cells were implanted into each flank of the six mice. (b) Representative images of tumors. (c) Tumor growth curve, *n* = 5 per group; the error bars represent SEM (d) Immunohistochemistry analysis of Ki67 expression and quantification of TUNEL^+^ cell per field in tumor tissues (*n* = 3 mice per group). (e) Representative images of indicated staining in (d). (f) Mice body weights of HeLa and HeLa/ADR groups. (*P < 0.05, **P < 0.01, and ***P < 0.001).

To verify the fitness differences between HeLa cells and HeLa/ADR cells under microenvironmental constraints, HeLa cells were implanted in the right flank of Nu/Nu mice (*n* = 5) and HeLa/ADR cells were implanted in each flank of Nu/Nu mice (*n* = 6). Initially, we observed that significantly fewer HeLa/ADR cells grew in mice compared with their parental cells (Figure 3a), and the progression of HeLa tumors was apparently faster than that of HeLa/ADR tumors (Figure 3b and c). Further study demonstrated that HeLa/ADR tumor cells had a much slower proliferation rate than the parental tumor cells based on Ki67 staining (Figure 3d and e). There was no significant difference in apoptotic cells between HeLa/ADR tumor cells and HeLa tumor cells based on a TUNEL assay (Figure 3d and e). These results demonstrated that HeLa/ADR cell lines exhibited remarkably reduced proliferation *in vivo*.

### 3.4 HeLa cells can completely suppress the growth of HeLa/ADR cells *in vivo*

**Figure 4:**
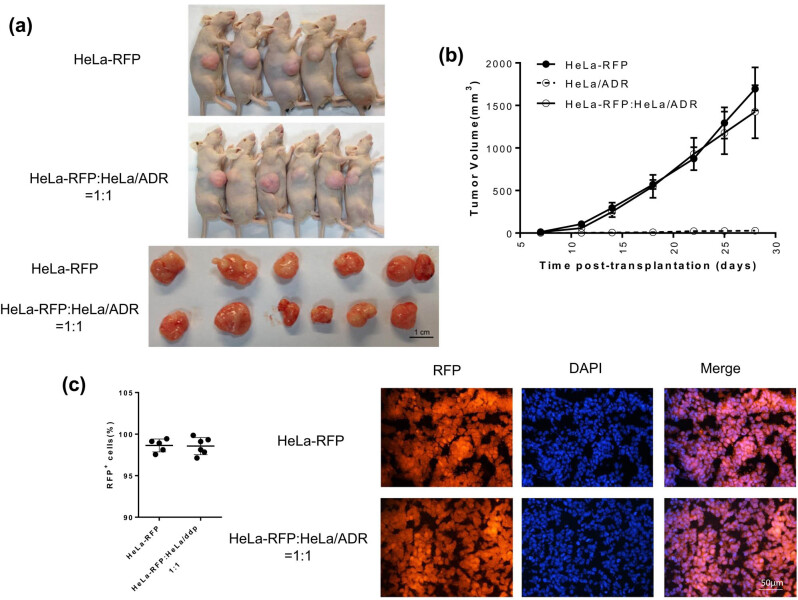
The growth of ADR-resistant cells is significantly inhibited by ADR-sensitive cells *in vivo.* (a) Images of tumors. (b) Tumor growth curve, *n* = 5 for HeLa group and *n* = 6 for mixed group; the error bars represent SEM (c) Analysis of RFP-positive cell per field; each dot indicates a tumor. (*P < 0.05, **P < 0.01, and ***P < 0.001).

To evaluate the interaction between HeLa and HeLa/ADR cell lines when they coexist *in vivo*, we formed tumors that consisted of RFP-tagged HeLa cells and HeLa/ADR cells at an equal initial proportion to promote cooperation or competition. Although mixed groups had double the number of initial cells, no significant difference in tumor growth was observed between these two groups (Figure 4a and b), indicating that neither HeLa cells nor HeLa/ADR cells increased the growth of mixed tumors. Then, we analyzed the percentages of RFP-positive cells in these two groups when the mice were killed, and there was no significant difference in the proportion of RFP-positive cells between mixed groups and HeLa cell groups (Figure 4c), demonstrating that the development of HeLa/ADR cells was fully suppressed by the growth of HeLa cells. Together, our results implied that the development of HeLa/ADR cells was significantly slower than that of HeLa cells and was fully inhibited when HeLa/ADR cells coexisted with HeLa cells *in vivo*.

## 4 Discussion

The solid tumor microenvironment has a dramatic effect on tumor development. Limited resources and architecture of the microenvironment restrict the volume of solid tumors at every period of their progression [10]. Tumor growth will slow down as it becomes larger following the Gompertzian growth. The tumor cell doubling time (approximately 1–2 days) is substantially faster than the tumor volume doubling time (approximately 60–200 days) [15], indicating that most tumor cells either die before they can divide or remain dormant within the tumor microenvironment. Thus, natural selection in tumors occurs through competition for nutrition and space [10], and the most-fit clone will defeat other clones.

Intratumor heterogeneity is a common feature of advanced cancers because of genomic instability within tumors [16,17,18,19], and a diverse cell population will be generated during tumor progression in this context [20]. The aim of current antitumor therapy strategies is to eradicate the maximum number of tumor cells, but variable responses of tumor subclones to different environmental pressures during various phases of tumor development produce tumors with potential ability to adapt to cytotoxic treatment, complicating tumor eradication [21]. However, adaptive therapy can significantly prolong patients’ overall survival by utilizing competitive relationships among different subclones, instead of maximizing cell killing [11,12]. Adaptive therapy is based on the theory that drug-sensitive cells are more fit than drug-resistant cells without drug treatment because resistant cells need to maintain the resistance mechanism to continue functioning, even in the absence of the drug pressure [22]. For example, resistance mechanisms involve a series of biosynthetic processes that require NADPH consumption, including the suppression of apoptosis in toxic conditions and enhanced antioxidant capacity. Meanwhile, cell proliferation also includes a series of processes that require NADPH consumption, including the biosynthesis of amino acids, fatty acids, and nucleotides. However, NADPH availability is limited for these processes, and if NADPH is increasingly used to maintain resistance mechanisms, the activity of anabolic processes will be restricted, further hindering proliferation [23,24]. In our study, we noticed that ADR-sensitive cells’ growth was significantly faster than that of ADR-resistant cells, even when nutrients were abundant, indicating that ADR-resistant cells require many resources for drug resistance processes, impacting proliferation. Additionally, our results demonstrated that ADR-sensitive cells could completely inhibit the development of ADR-resistant cells *in vivo*. We inferred that if certain quantities of ADR-sensitive cells are maintained during ADR treatment in cervical cancer, sensitive cells may inhibit the development of ADR-resistant cells by competition for nutrition and space, and accordingly, may delay the development of ADR resistance.

In the previous study, ADR-resistant breast cancer cells did not display apparent defect in the abundance of glucose, but showed fitness deficits under energy-restricted conditions compared with sensitive cells, and patient survival time could be extended by adaptive therapy based on a computational model [14]. In our study, the HeLa/ADR cell line exhibited a significant fitness deficit, even in optimum conditions, and had notably slower growth *in vivo*, indicating that the ADR-resistant mechanism has diverse impact on the growth of various tumors. Accordingly, adaptive therapy may have specific efficacies depending on tumor type. Additional studies are needed to identify the types of tumors susceptible to ADR-resistant mechanisms to develop more precise, individualized adaptive therapies.

## References

[1] Yang F, Teves SS, Kemp CJ, Henikoff S. Doxorubicin, DNA torsion, and chromatin dynamics. Biochim Biophys Acta. 2014;1845(1):84–9. 10.1016/j.bbcan.2013.12.002 PMC3927826 24361676

[2] Goncalves M, Mignani S, Rodrigues J, Tomas H. A glance over doxorubicin based-nanotherapeutics: from proof-of-concept studies to solutions in the market. J Control Rel. 2020;317:347–74. 10.1016/j.jconrel.2019.11.016 31751636

[3] Landau DA, Carter SL, Stojanov P, McKenna A, Stevenson K, Lawrence MS, et al. Evolution and impact of subclonal mutations in chronic lymphocytic leukemia. Cell. 2013;152(4):714–26. 10.1016/j.cell.2013.01.019 PMC3575604 23415222

[4] Landau DA, Tausch E, Taylor-Weiner AN, Stewart C, Reiter JG, Bahlo J, et al. Mutations driving CLL and their evolution in progression and relapse. Nature. 2015;526(7574):525–30. 10.1038/nature15395 PMC4815041 26466571

[5] Schwarz RF, Ng CK, Cooke SL, Newman S, Temple J, Piskorz AM, et al. Spatial and temporal heterogeneity in high-grade serous ovarian cancer: a phylogenetic analysis. PLoS Med. 2015;12(2):e1001789. 10.1371/journal.pmed.1001789 PMC4339382 25710373

[6] Dagogo-Jack I, Shaw AT. Tumour heterogeneity and resistance to cancer therapies. Nat Rev Clin Oncol. 2018;15(2):81–94. 10.1038/nrclinonc.2017.166 29115304

[7] Ramirez M, Rajaram S, Steininger RJ, Osipchuk D, Roth MA, Morinishi LS, et al. Diverse drug-resistance mechanisms can emerge from drug-tolerant cancer persister cells. Nat Commun. 2016;7:10690. 10.1038/ncomms10690 PMC4762880 26891683

[8] Hata AN, Niederst MJ, Archibald HL, Gomez-Caraballo M, Siddiqui FM, Mulvey HE, et al. Tumor cells can follow distinct evolutionary paths to become resistant to epidermal growth factor receptor inhibition. Nat Med. 2016;22(3):262–9. 10.1038/nm.4040 PMC4900892 26828195

[9] Das TM, Salangsang F, Landman AS, Sellers WR, Pryer NK, Levesque MP, et al. Modelling vemurafenib resistance in melanoma reveals a strategy to forestall drug resistance. Nature. 2013;494(7436):251–5. 10.1038/nature11814 PMC3930354 23302800

[10] Greaves M, Maley CC. Clonal evolution in cancer. Nature. 2012;481(7381):306–13. 10.1038/nature10762 PMC3367003 22258609

[11] Gatenby RA, Silva AS, Gillies RJ, Frieden BR. Adaptive therapy. Cancer Res. 2009;69(11):4894–903. 10.1158/0008-5472.CAN-08-3658 PMC3728826 19487300

[12] Enriquez-Navas PM, Kam Y, Das T, Hassan S, Silva A, Foroutan P, et al. Exploiting evolutionary principles to prolong tumor control in preclinical models of breast cancer. Sci Transl Med. 2016;8(327):324r–27r. 10.1126/scitranslmed.aad7842 PMC4962860 26912903

[13] West J, You L, Zhang J, Gatenby RA, Brown JS, Newton PK, et al. Towards multi-drug adaptive therapy. Cancer Res. 2020;80(7):1578–89. 10.1158/0008-5472.CAN-19-2669 PMC7307613 31948939

[14] Silva AS, Kam Y, Khin ZP, Minton SE, Gillies RJ, Gatenby RA. Evolutionary approaches to prolong progression-free survival in breast cancer. Cancer Res. 2012;72(24):6362–70. 10.1158/0008-5472.CAN-12-2235 PMC3525750 23066036

[15] Klein CA. Parallel progression of primary tumours and metastases. Nat Rev Cancer. 2009;9(4):302–12. 10.1038/nrc2627 19308069

[16] Gerlinger M, Rowan AJ, Horswell S, Larkin J, Endesfelder D, Gronroos E, et al. Intratumor heterogeneity and branched evolution revealed by multiregion sequencing. N Engl J Med. 2012;366(10):883–92. 10.1056/NEJMoa1113205 PMC4878653 22397650

[17] Wang Y, Waters J, Leung ML, Unruh A, Roh W, Shi X, et al. Clonal evolution in breast cancer revealed by single nucleus genome sequencing. Nature. 2014;512(7513):155–60. 10.1038/nature13600 PMC4158312 25079324

[18] de Bruin EC, McGranahan N, Mitter R, Salm M, Wedge DC, Yates L, et al. Spatial and temporal diversity in genomic instability processes defines lung cancer evolution. Science. 2014;346(6206):251–6. 10.1126/science.1253462 PMC4636050 25301630

[19] Gerstung M, Jolly C, Leshchiner I, Dentro SC, Gonzalez S, Rosebrock D, et al. The evolutionary history of 2,658 cancers. Nature. 2020;578(7793):122–8. 10.1038/s41586-019-1907-7 PMC7054212 32025013

[20] Burrell RA, McGranahan N, Bartek J, Swanton C. The causes and consequences of genetic heterogeneity in cancer evolution. Nature. 2013;501(7467):338–45. 10.1038/nature12625 24048066

[21] Nowell PC. The clonal evolution of tumor cell populations. Science. 1976;194(4260):23–8. 10.1126/science.959840 959840

[22] Aktipis CA, Boddy AM, Gatenby RA, Brown JS, Maley CC. Life history trade-offs in cancer evolution. Nat Rev Cancer. 2013;13(12):883–92. 10.1038/nrc3606 PMC4010142 24213474

[23] Jerby L, Wolf L, Denkert C, Stein GY, Hilvo M, Oresic M, et al. Metabolic associations of reduced proliferation and oxidative stress in advanced breast cancer. Cancer Res. 2012;72(22):5712–20. 10.1158/0008-5472.CAN-12-2215 22986741

[24] Schafer ZT, Grassian AR, Song L, Jiang Z, Gerhart-Hines Z, Irie HY, et al. Antioxidant and oncogene rescue of metabolic defects caused by loss of matrix attachment. Nature. 2009;461(7260):109–13. 10.1038/nature08268 PMC2931797 19693011

